# Together for better health in Germany. Towards a public health strategy

**DOI:** 10.1093/eurpub/ckac131.536

**Published:** 2022-10-25

**Authors:** S Matusall

**Affiliations:** Zukunftsforum Public Health, Berlin, Germany

## Abstract

In Germany, the responsibility for public health is divided both between federal levels and between sectors, and there is no national or general strategy for public health. However, a strategy is essential for achieving the overarching goal of ensuring the best possible health for all people, including preventing and addressing significant health challenges such as pandemics and climate change.

In 2016, Zukunftsforum Public Health (Future Forum Public Health) was established with the primary goal to enhance coherence and collaboration between public health stakeholders from academia, policy and practice. According to the principle of health in all policies, it also aims to promote collaboration across federal levels and political sectors. Within the platform provided by Future Forum, the public health community agreed that a strategy was necessary for strengthening and uniting the disparate field of German public health even though no official mandate for developing a strategy existed. Thus, Future Forum launched a grassroots process to identify key elements for a strong and resilient public health system, based on the essential public health operations (EPHOs), as defined by WHO Europe. For each of the ten EPHOs, the key issues paper “Towards a Public Health Strategy - Together for Better Health in Germany” presents a description of the current situation and challenges, an introduction to the key players, a definition of the aims and objectives, and concrete proposals on how to achieve these. Through its participative nature, the process of developing the key issues itself was an important factor for strengthening the public health community. This process highlights that even where national public health strategies and structures are missing, grassroots public health movements can be effective in strengthening the public health community as well as producing a visible set of recommendations for a resilient public health system.

**Key messages:**

• Based on WHO’s essential public health operations, Future Forum Public Health developed a key issues paper with guidelines for a resilient public health system.

• The participative process leading to the key issues paper was itself an important factor for networking and strengthening Germany’s public health community.

